# Thomas W. Kensler (1948–2025): A Legend in Antioxidant Response Pathways and Aflatoxin Carcinogenesis Research

**DOI:** 10.3390/toxins17100517

**Published:** 2025-10-21

**Authors:** David L. Eaton, John D. Groopman

**Affiliations:** 1 Department of Environmental and Occupational Health Sciences, School of Public Health, University of Washington, Seattle, WA 98105, USA; 2 Department of Environmental Health & Engineering, Bloomberg School of Public Health, The Johns Hopkins University, Baltimore, MD 21205, USA


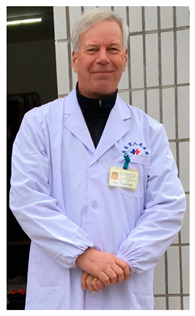


It is with great sadness that we must include in this Special Volume of *Toxins* a memorial to our close friend and colleague, Dr. Thomas W. Kensler, who passed away from a hiking accident on Mt. Blanc, France, on 11 July 2025, at the age of 76. Dr. Kensler was born in New York City in 1948, the son of Charles J. Kensler and Elizabeth B. K. Williams. He grew up in Lexington, Massachusetts. After earning his AB in biology from Hamilton College, he then received his PhD in toxicological sciences from the Massachusetts Institute of Technology in 1976, in the laboratory of Dr. Gerald Wogan. He then completed postdoctoral training at the McArdle Laboratory for Cancer Research at the University of Wisconsin and at the National Cancer Institute in cancer therapeutics before joining the faculty of the Johns Hopkins Bloomberg School of Public Health in 1980. Tom spent 30 years at Hopkins before he and his wife, Dr. Nancy Davidson, moved to the University of Pittsburgh in 2010 (he maintained his appointment and collaborations with his colleagues at Hopkins Bloomberg School of Public Health). In 2017, he and Dr. Davidson moved to Seattle where Nancy was appointed as President and Executive Director of the Seattle Cancer Care Alliance and Senior Vice President at Fred Hutchinson Cancer Center and Tom continued his research in the Public Health Sciences Division for 7 more years, retiring in 2024. Dr. Kensler was a giant in several related areas in toxicology, including the toxicology of aflatoxins, on which this Special Issue of *Toxins* is focused. Over his nearly 50 years of research, Dr. Kensler published nearly 500 research papers, book chapters and reviews, of which 400 were published in peer-reviewed journals.

His research has been cited by others over 45,000 times, giving him an “H-Index” of 109, which is remarkable (Figure 1) One online source states that “for top-tier professionals, H-indices greater than 40 are generally considered excellent, and 60 or higher may be considered remarkable or exceptional”. Tom mentioned to one of us many years ago, “My goal is to have an H-factor higher than my age when I die.” He more than met that goal! This is itself an incredible testament to the scientific impact that Dr. Kensler has had in toxicology and more specifically cancer prevention research.

Tom was a major contributor to the field of cancer chemoprevention, with much of his work focused on novel approaches to dietary modulation of aflatoxin biotransformation [1,2,3,4,5,6]. His contributions in the area focused on prevention of dietary aflatoxin-induced liver cancer, yet only 25% of his research publications were related to aflatoxins. He made significant contributions in the broader (albeit related) area of ‘redox biology’, with a focus on understanding how certain vegetable components in the diet can modify gene expression of antioxidant pathways that help protect against oxidative stress [7,8,9]. His research was both fundamental (e.g., at the molecular level) and directly applicable to ‘real-world’ cancer prevention. His elegant studies on the Keap1-Nrf2 antioxidant pathway and its modification by dithiolthiones (including sulforaphane found in broccoli and other cruciferous vegetables) led the way for direct human intervention studies in populations at increased risk for certain cancers [10,11,12,13,14,15,16,17].

Despite the molecular details of much of his research, he never lost sight of the bigger picture and was an outstanding collaborator with many clinicians interested in research that saves lives. For example, in 2016, he was first author on a review titled “Transforming Cancer Prevention through Precision Medicine and Immune-oncology” [18]. The 11 co-authors (including his wife, Nancy) were leading cancer prevention researchers from 12 of the most prestigious cancer research and treatment centers in the United States. In this article, the authors note “Just as precision medicine and immune-oncology are revolutionizing cancer therapy, these approaches are transforming cancer prevention. Here, we set out a brief agenda for the immediate future of cancer prevention research (including a “Pre-Cancer Genome Atlas” or “PCGA”), which will involve the inter-related fields of precision medicine and immunoprevention—pivotal elements of a broader domain of personalized public health.”

Tom’s contributions to cancer prevention research were widely recognized by his colleagues and by major professional organizations. For example, in 2007, he was honored by the American Association for Cancer Research and the American Cancer Society through with the annual “Award for Research Excellence in Cancer Epidemiology and Prevention”. Two years later in 2009, the Society of Toxicology recognized Tom with one of their highest awards, the Translational Impact Award. His research had a global impact and was greatly appreciated in China. In 2011, he received a National Friendship Award in Beijing, People’s Republic of China, which is the highest award for foreign civilians, and was made an honorary Professor at the Nantong Tumor Institute, Nantong, China, in 2015.

In addition to his remarkable contributions to cancer prevention research, Tom was an outstanding mentor and educator. Over his 40+ years in academia, he served as mentor/advisor for 28 doctoral students, 16 postdoctoral fellows, and 8 master’s degree students. He also served on over 120 other graduate student committees, alongside conducting viva voce exams and performing other mentoring roles. But his mentorship and influence on other scientists extend well beyond the hundreds of graduate students and postdocs he directly mentored. For example, Dr. Donna Zhang, a full Professor in Pharmacology and Toxicology at the University of Arizona, was quoted in Tom’s obituary in the Baltimore Sun: “His quiet brilliance, deep integrity, and unwavering generosity shaped not only the field of redox biology, but also my own journey in science.” We know that this perspective is shared by all who have known and worked with Tom over the years. As a testament to Tom’s passion to be a ‘good mentor, one of our colleagues shared this story: “I recall being with him at Hopkins as a visiting scientist. I attended a student seminar being presented by one of his students. Tom was really grilling the students to a point that it was difficult to see why he was being so critical. Patricia Egner [Tom’s senior lab tech] explained afterwards that Tom wanted his students to go out into the world, be respected from the first sentence they shared, be accurate with their sharing, and think about the bigger issues. It was better that life was uncomfortable for them in a safe environment (the seminar) than when they were out in the real world.”

He will be greatly missed as a dear friend and colleague, but we find solace that his tremendous work in the field will continue to inspire new research and save lives, and that—as tragic as his accident was—he left us doing something he loved.

Tom is survived by his wife of 43 years, Nancy E. Davidson, son Kevin H. Kensler, daughter Caroline B. Kensler and son-in-law Nikolas F. Iubel, brother-in-law and sister-in-law David L. Davidson and Kathy Reigstad, nephews David M. Davidson and Keith L. Davidson, and stepsisters Aimée Margolis and Nadia Margolis.

Contributions in Dr. Kensler’s memory can be made to the American Association for Cancer Research at www.aacr.org (accessed on 15 October 2025).

## Figures and Tables

**Figure 1:**
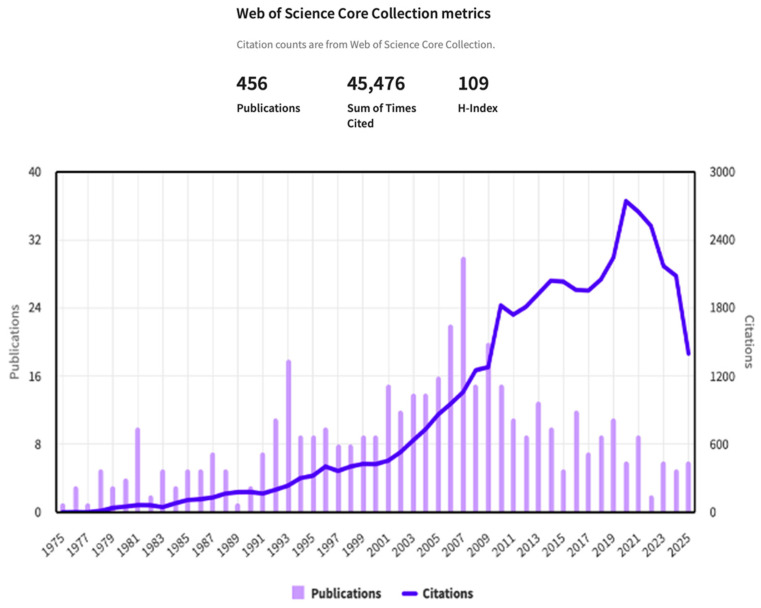
Web of Science Publications and Citations of Dr. Thomas Kensler 1975–2025.
